# Hospital and Institutional News

**Published:** 1915-12-11

**Authors:** 


### HOSPITAL AND INSTITUTIONAL NEWS.

### A FOREWORD TO OUR READERS.

Next week's issue of The Hospital being our Special Christmas Number should have a wide-spread value and interest for all workers in and supporters of our hospitals. It will be fully illustrated, and contain much which should produce a greater yield of income, a lessened expenditure, and increased efficiency in every intelligently administered voluntary hospital. The increasing demand for The Hospital and the unique and novel attractions of our Special Christmas Number in 1915 may make it desirable that copies should be ordered in advance to prevent disappointment.

### THE UNIFORM OF TRAINED NURSES.

In the *Nursing Mirror* of the 4th instant an article appears to have created general satisfaction throughout the nurse-training schools, for it offers a solution of the difficulties which have hitherto surrounded the problem of providing protection for the uniform of trained certificated nurses. The *Nursing Mirror's* suggested solution is the establishment of a standard uniform. The War Office, having taken the first step to protect the uniform of certain workers for the sick in their employ, cannot refuse to complete the good work by instituting a uniform to be worn on service by all certificated trained nurses working under their administration. So far as civil hospitals and certificated trained nurses in civil employment are concerned, the nurse-training schools might properly agree to the selection and adoption of one approved uniform to be worn only by certificated trained nurses. Then each training school and hospital could adopt its own special shoulder-straps, official number, and brassard, whilst the nurses trained at each hospital and school should have a common registration or regimental number to be worn with the certificated trained nurse's standard uniform. By this simple means the present difficulties in the way of securing protection, the chief of which is the fact that there are some 400 different uniforms at present worn by certificated trained nurses, would be removed. Every certificated trained nurse could then at once be given legal protection for her standard uniform, with its official straps, numbers, and brassards. We should welcome the opinion and support of all matrons, sisters, and trained nurses for this simple plan, which merits unanimous adoption and can so secure immediately the desired end.

### ST. GEORGE'S HOSPITAL'S WAR WORK AND NEEDS.

The governors of St. George's Hospital allocated, not long after the outbreak of war, 100 beds in their institution for the use of the Navy and Army. According to information supplied by the secretary, up to November 30 there have been 508 admissions from the seat of war—namely forty-eight sailors and 460 soldiers. Of the soldiers the great majority (412) were received from France, and the remainder from the Mediterranean; ten of them were Australians, and three were Canadians. Only two deaths have occurred, although some of the cases have been very severe ones, involving protracted treatment. About a hundred of the patients have had the further advantage of admission to the Atkinson Morley Convalescent Hospital at Wimbledon, the well-known adjunct of St. George's. When the war began, St. George's was for a time largely denuded of its staff, and it was only by the patriotic help of various old St. George's men that the work of the hospital was kept going and the interests of the patients conserved. All difficulties were satisfactorily surmounted, and the committee are to be congratulated on the way in which they have risen to the national emergency. The secretary will be glad to receive presents of tobacco, cigarettes, and game for the sailors and soldiers in the hospital at Christmas.

### A "CAVELL" WARD IN A NEW HOSPITAL.

The Liverpool Invalid Children's Association is building a hospital for children to be known as the Leasowe Hospital for Children, and it has been decided to provide a memorial to Edith Cavell in the form of a twenty-two bed ward. The building scheme of the new hospital comprises four blocks of open-air wards, each of which accommodates forty-four beds. Two Liverpool citizens have been commemorated in the case of two of these blocks, which have been occupied by patients since they were opened by Lord Derby in July 1914. The remaining two blocks are being built. The "Cavell" ward is to be in one of these blocks known as "Honour." When complete the hospital will accommodate from 200 to 250 beds, and is expected to cost £48,000. Of this sum £12,000 has been already subscribed apart from a grant from the Treasury of £18,000, so that only £10,000 now remains to be raised. A donation of £200 is sufficient to secure the naming of a bed in the "Honour" block after officers or men who have fallen in the war.

### A HEALTHY HOSPITAL SUNDAY FUND.

It has not been often of recent years that we have been able to record a substantial and steady increase in the sum total received from Hospital Sunday in this or that provincial city. It is all the more pleasing, therefore, to see the healthiness of the Hospital Sunday Fund in Manchester. The Fund is in the remarkable position of possessing an ordinary income which is the largest amount received for twenty-four years. Not only did the total amount surpass that of 1914 by £632, which might have been due to a chance subscription, but the number of contributing churches was larger by 135 and almost twice as large as the number which contributed to the fund in 1913. Considering the efforts which Manchester has made in other directions, notably, of course, for Red Cross and war work of different kinds, this is very satisfactory. It also shows the vitality of the voluntary system, since the war, which might have been fancied by some to threaten its position, has actually rallied popular support, and left our hospitals, on the whole, in a stronger position than ever. There are few better barometers of this vitality than the condition of the great funds. For as previous to the war the Hospital Sunday Fund movement showed some signs of going to sleep, we may hope that the war will prove, in other centres besides Manchester, a stimulus and a cause of re-awakening. It should be noted, however, that the remarkable increase in the number of contributing churches, which rose from 472 in 1913 to 730, points a criticism to the fund's administration in the past. Other cities where the Sunday Fund yields poor results would, therefore, be well advised to examine the proportion of contributing churches to the actual places of worship. Where the fund makes little or no progress the strong probability is that many churches do not contribute at all.

### PRESENTATIONS AT A SOMERSET V.A.D. HOSPITAL.

The Countess Temple made some interesting presentations to certain members of the staff of the Voluntary Aid Hospital, Newton Park, Somerset, on the anniversary of its opening last week. On November 30, 1914, the hospital was opened with twenty-five beds, and shortly afterwards a theatre was added. Later ten more beds were installed, and so far 128 patients have passed through the building. After Lady Temple had presented a silver cigarette case to Mr. L. D. Beath, M.R.C.S., the medical officer in charge, and to Mr. F. Sprawson, the commandant, and a gold bracelet and a brooch to the matron and the senior probationer, the Countess herself received a presentation from the staff and patients "as a slight token of respect on the first anniversary" of the opening of the hospital. The gift to Lady Temple took the form of a silver-mounted coffee-stand.

### MEDICAL ADVERTISEMENTS AND THE ARMY.

In view of the very definite Ministerial pronouncement in the House of Commons on the subject of advertisements which may interfere with recruiting, the *British Medical Journal* has decided that until further notice no advertisement will be accepted for insertion if it appears that it may result in the relegation to civil practice of any practitioner who, in view of the national emergency, should be serving with the Colours. In other words, all advertisers of vacant posts, practices for transfer, and so forth, must make it clear that only applications from those who are ineligible for military service will be dealt with. This will, of course, result in greater difficulty than ever for voluntary hospitals and for those medical men who wish to sell practices or engage *locum tenentes*; but *à la guerre comme à la guerre*, and we believe the Association's action is certain to command the approval of the profession at large. For ourselves, with the *British Medical Journal*, we propose as far as possible to follow this course in the letter and in the spirit.

### A RED CROSS CONFERENCE AT STOCKHOLM.

Official announcements are reported by Reuter concerning a Red Cross Conference held at Stockholm between Russian, German, and Austro-Hungarian delegates. All the decisions relating to the care of prisoners of war are understood to have been unanimous, and it is recommended that a mixed commission should be set up, consisting of three neutral members and six others, to visit internment camps of the belligerents, and to issue reports to the authorities on any irregularities that might be observed.

### THE ALTON CRIPPLES HOME.

Sir Wm. Treloar has issued an eloquent appeal on behalf of the institution which he was the means of founding, and with which his name is inseparably connected. The income of this admirable charity is never sufficient to meet all the calls for help which are received from the crippled children, or rather from those responsible for them. There are in the hospital at present two hundred and fifty crippled children, drawn from all parts of the country; and in the college another sixty crippled boys are receiving a technical training. There is always a lengthy waiting list, and any curtailment in the income of the charity, whether owing to the war or to any other cause, must inevitably mean the lengthening of that pathetic list, and the postponement of treatment for many to whom its early initiation may mean the difference between life and death. The splendid work done at Alton is too well known to need commendation in these columns: there are few worthier voluntary hospitals in England, and we sincerely trust that the liberal support which it has had in the past will not fail now or in the future.

### THE MAUDSLEY HOSPITAL FOR WAR PURPOSES.

The Army authorities have expressed to the London County Council a desire to take over and equip the Maudsley Hospital buildings, which are now practically complete, for use in connection with the adjacent 4th London General Military Hospital. The Mental Hospitals Committee of the Council have consented to this, having obtained the generous concurrence of Dr. Henry Maudsley in the postponement of the use of the buildings for the specific purpose for which he originally contributed to their cost, and which the committee know to be a matter of the deepest interest and concern to him. Osnabruck House, which was acquired by the Council for use as a nurses' home in connection with the Maudsley Hospital, has already been furnished and has received military patients. It is understood that any extra expense entailed upon the Council by alterations in the building arrangements will be made good by the Government.

### THE LEAGUE OF MERCY.

We have received a notice from the honorary secretaries concerning the annual meeting of the League of Mercy, which omits to mention the date, place, and hour of meeting. The notice refers to one of the most important occasions that has occurred in its history. It intimates that the Right Hon. James Lowther, Speaker of the House of Commons, who has shown the deepest and most knowledgeable interest in the voluntary hospitals, who is rendering continuous personal service as one of the governors of the King's Hospital Fund, and who is one of the most forcible and convincing speakers of our time, will give an address at the annual meeting. This will be held at St. James's Palace on Tuesday, December 21, at 3 P.M. Mr. Lowther should have a crowded and enthusiastic audience, and his address cannot fail to quicken public interest in the voluntary hospitals. At the same meeting H.R.H. Princess Alexander of Teck will present the crosses of the Order of Mercy awarded during the past year.

### CEREBRO-SPINAL FEVER IN AUSTRALIA.

Considering the differences of opinion in this country concerning the value of Flexner's serum for cerebro-spinal meningitis, it is of interest to note the experience of the medical profession during the epidemic which is now in progress in Australia. This epidemic, it may be remarked, reached its height about the middle of October, and by the last reports it is now showing signs of abating. Victoria has suffered more than the other provinces, having had 470 cases, with 34 per cent. of deaths. The American serum is said to have been very unsatisfactory, which confirms the views prevalent in the Navy and among some, though not all, of the Army medical officers. The continuance of the epidemic is alleged to be due to the number of conflicting authorities, whose efforts overlap and neutralise one another. New South Wales, which has been but little affected, is now clear of the disease; but a fresh outbreak is reported in Tasmania.

### TROUBLE AT EDMONTON.

The Edmonton Urban District Council has adopted a vote of censure upon the medical officer of health, Dr. S. C. Lawrence, and has made an attempt to edit his annual report. This is not the first time that the Council has made an endeavour to get rid of its medical officer of health, and the proceeding has aroused much indignation among his professional colleagues. According to the *Medical Officer*, Dr. Lawrence is accustomed to be "honestly outspoken in his comments" upon sanitary affairs in Edmonton, and has thus incurred the hostility of certain powerful vested interests. It is, of course, fatal to efficiency in the Public Health Service if the Local Government Board is to see only edited versions of expert opinion on the sanitation of each district, bowdlerised to suit the views of interested parties; and if the Edmonton Council really have any reasonable grounds for dissatisfaction with Dr. Lawrence, they are putting themselves in the wrong by their method of expressing it. As to the merits of the case, we offer no opinion except that it is up to the Edmonton Council to refute, if it can, the indictment drawn up by the *Medical Officer*.

### MISS BUCKINGHAM OF BIRMINGHAM.

A most useful and helpful hospital matron has been lost to British nursing by the rather tragic death of Miss Maude A. Buckingham, matron of the Queen's Hospital, Birmingham. Miss Buckingham was for a number of years organising matron of the Nursing Service of the Territorial Force, Warwickshire and Worcestershire. She rendered good service in connection with the organisation of the details of the nursing department of the 1st Southern General Hospital at Bournbrook, and at the time of her death was in charge of the Hollymoor War Hospital, Birmingham. Miss Buckingham was trained at St. Bartholomew's Hospital, was then appointed sister at Scarborough, then matron at Rotherham Hospital and Dispensary, and then in March 1907 matron of the Queen's Hospital, Birmingham. At this latter institution she found a most congenial field of labour, and showed her administrative gifts by organising, on the extension of this hospital, three new medical wards. She was much appreciated by her nurses and staff, who will miss her sadly and will, we fear, miss her counsel not once but often in their nursing career. She founded the Queen's Hospital Nurses' League, of which she became the first president, and was a prominent member of the committee of the Trained Women Nurses' Friendly Society. Our correspondent in Birmingham testifies to the keenness of the grief caused by her death, which will entail serious loss upon the Queen's Hospital, Birmingham, and is deeply regretted by all the members of the staff, whose trusted and sincere friend she has so often proved herself to be. The interment took place at Kensal Green, London, on December 9. A memorial service, conducted by the Bishop of Birmingham, was held in the chapel of the Queen's Hospital, Birmingham, on the same day.

### THE WELSH MEDICAL SCHOOL.

We have already adverted on recent occasions to the refusal of the Treasury to consent to the building programme of the Welsh Medical School at Cardiff, owing to the dissatisfaction of the Whitehall authorities with the response to their demands for reorganisation of the University of Wales. In particular, they have refused permission for Sir W. J. Thomas's munificent schemes to be carried out. At a meeting of the members of the Welsh University Education Conference held in Shrewsbury lately, Mr. Ingledew and Colonel Bruce Vaughan spoke very strongly in favour of a resolution which earnestly urged upon the Treasury the need for leave to proceed with the medical school buildings, promising that the building operations should be so conducted as not to afford employment to men available for military service and to men who would otherwise be rendering war service. It is certainly hard on the hospital and medical school authorities at Cardiff that their much-needed buildings, the money for which had already been provided before the war began, should be prohibited just because the University of Wales is somewhat recalcitrant. The medical school will be, of course, part of the University in the end; but pressure can be, and is being, put on the University leaders in other ways, without penalising the unfortunate school, which is not at all immediately concerned in the quarrel. We trust Colonel Bruce Vaughan's advocacy will melt the hearts of the Whitehall authorities.

### THE SEX QUESTION IN MEDICAL APPOINTMENTS.

The question whether a man or a woman doctor should fill the post of medical officer to the Bath Baby Clinic has been the cause of much controversy in Bath, where the dispute has been aggravated by a discussion, after a gentleman had been appointed, of the successful candidate's eligibility for military service. It appears that the Bath Sanitary Committee recently appointed Dr. Welch as part-time medical officer to the Bath Childbirth and Infant Welfare Clinic, but that at a subsequent meeting of the City Council it was decided to refer back the minute of the committee which reported the appointment, on the ground that the committee's impression that Dr. Welch was ineligible for military service had since been discovered to be incorrect. Medical women, it was stated, had previously been in charge of the work; the advertisement contained no clause excluding men, and a man had been selected. It was therefore argued that to rescind the appointment was unfair to the successful candidate and insulting to the profession. On the Chairman stating his belief that it would very soon be determined what men were required for the country's service, it was eventually decided to drop the matter temporarily.

### THE DAY ROOM REVIVAL.

Before the war the day room which used to form an adjunct to the ordinary hospital ward was fast being done away with, mainly owing to the desire to empty the beds as fast as possible and to remove patients at the earliest possible date to convalescent homes. With the opening of military hospitals, not always filled with very serious cases, and often acting as semi-convalescent homes, the need for day rooms, or recreation huts as they are called, is once more in evidence. Thus two of the military hospitals of Plymouth have been presented with huts of this kind, a condition of the gift in each case being that the hut should be managed by a committee of soldiers. Major-General Penton, who presided at the opening ceremony of the second hut, declared that the hut should be an example to every hospital. It is clear, however, that the need for them in the military hospitals is no indication that they will be considered necessary in ordinary hospitals once normal conditions return.

### INFANT WELFARE CENTRES IN SUSSEX.

The movement for the establishment of child welfare centres and clinics in Sussex is now strengthened by the adherence of Haywards Heath to a scheme, for the merit of founding which the Haywards Heath, Lindfield, and Scaynes Hill Nursing Association is responsible. A provisional committee started work before calling a public meeting to endorse and support it, and three rooms have been secured, with Dr. Killpack as medical officer. That the movement is no sporadic effort of enthusiasm may be judged from the fact that the services have been secured of Miss Rose, who, as assistant superintendent of the County Nursing Association, has been appointed to organise child welfare centres under the Public Health Department of the County Council. The provisional committee estimate the expenses at £40 per annum, towards which the Board of Education will contribute a grant if the standard of work be approved. The Chairman declared at the inaugural meeting that the only free thing which the mothers would get was advice. In an address given by Miss Holford, secretary of Infant Consultations and Schools for Mothers, it was stated that 600 such centres were now at work, and that 200 of these had been established since the outbreak of war. The work began on December 9.

### CHILD PRESERVATION IN SCOTLAND.

The Scottish Midwives Bill has been introduced into Parliament, and read a second time. Nor is this the only evidence that Scotland is not standing where she did in infant preservation work; for there is considerably more activity in these matters going on than ever before. At a conference held recently in Glasgow, one of several arranged by the Scottish Insurance Commission between the maternity hospitals and the Scottish approved societies, attention was drawn by Dr. Jardine to the ante-natal department organised at the Glasgow Royal Maternity and Women's Hospital, and to their proposals for establishing a post-natal department also. We feel sure that nothing but good can come of educating the representatives of the wage-earners, through their approved societies, in the latest developments of child protection before, during, and after birth. Incidentally, Dr. Jardine quoted some wonderful facts concerning the practice of the hospital, which must surely act as a good advertisement for that excellent charity. On a day recently, Cæsarean section was performed three times within two hours within its walls; while in 1914 it was done four times in a day once, and three times in a day twice; in every case both mother and child were saved.

### A COMPLAINT AGAINST GUY'S.

At an inquest conducted lately by the Coroner for the City and Southwark, on an aged person who died in Guy's Hospital, some complaints were heard about the procedure of the residents at that institution in connection with inquests. In the particular case under review it seems from the report as if the blame for confusion and delay had rested partly with the practitioner who sent the case to hospital; but there certainly appeared on the part of the hospital staff some thoughtlessness and casualness in dealing with the case, and the Coroner was moved to declare that it does not stand alone. We feel sure that after this expression of opinion the resident staff will wake up. Not improbably the pressure of war service has resulted in a rather inexperienced set of office holders, who will doubtless speedily learn the wisdom of being civil to constituted authority.

### BRADFORD SELF-INDICTED.

Some interesting observations were made in the speeches which concluded the inspection of the lately opened Bradford War Hospital by Surgeon-General Kenny, C.B., of the Northern Command. For instance, General Kenny himself declared, with reference to his attempt nearly a year ago to start a military hospital in schools and one large house in Bradford, that houses and schools, after all, were not hospitals, and were built for very different purposes. It is good to have this statement on record from such a source, for The Hospital has often had to point out to local authorities, and even on occasion to military also, that schools, a rule to which there are very few exceptions, should never be used for hospital purposes. Another interesting statement was made by Mr. F. H. Bentham, who explained why Bradford had apparently lagged behind other cities in providing accommodation for the wounded. The war hospital scheme, he remarked, was the best that Bradford could do under existing circumstances. In Birmingham—about twice the size of Bradford—4,000 beds for soldiers had been organised. But in Bradford, Mr. Bentham added, there were not the facilities; and while "the medical equipment was sadly deficient, the hospitals were out of date." This meant in practice that St. Luke's Poor-Law Hospital was the only available institution in the city. The Board of Guardians therefore felt that it rested with them to retrieve the honour of Bradford. St. Luke's Hospital could accommodate 540 beds, and this number could be raised to 600, if necessary, later. This striking self-indictment, which apparently passed without comment, should be enough to arouse the citizens of Bradford to bring all their hospitals up to date. The above confession is lamentable, though the speaker deserves credit for his frankness.

### THE PORTSMOUTH EYE AND EAR HOSPITAL.

A certain amount of commotion appears to have been aroused locally by the refusal of the committee of this institution to adopt a rule, proposed by Colonel Charles Ford, that "no member of the committee shall, either himself or herself, or as a member of a firm or company, have any pecuniary interest in supplying goods or in any contract for the institution." It is pointed out that the rules of the Royal Portsmouth Hospital contain such a clause, and that no information is obtainable about the contractors and tenderers for the supplies needed by the Eye and Ear Hospital. The local Press has commented on the subject with some reserve, but is apparently in favour of the rule, which has proved salutary. The hospital can have nothing to lose by complete candour and publicity, without which it stands to fall in public estimation and should fail in attracting support. There may be reasons for the committee's refusal, but we doubt if they are as strong as those in favour of Colonel Ford's proposed reform, which is urgent.

### PHARMACISTS AS "INDISPENSABLES."

As we anticipated recently, pharmacists, after many applications and deputations to Government Departments, are now placed on the "starred" list of indispensable workers. In addition to those specially mentioned doing Insurance dispensing under the National Insurance Act, "chemists" apparently will include all pharmacists actively engaged in the various branches of pharmaceutical work. The position of institutional pharmacists would now appear to be that they should attest under the Derby scheme, at the same time notifying the recruiting officials that they are doing indispensable work. No doubt hospital and other institutional authorities will apply, in due course, for exemption for pharmacists and other trained workers in their employ whom it is impossible to replace. Lord Derby has agreed to arrange for cases to come up before the local tribunal nearest the head office of large concerns, and this would appear to be the most satisfactory procedure, for now governing bodies of our hospitals, etc., may have the position of their employees dealt with all together. It seems a pity that the excellent material available has not been made use of, and that still the Army authorities give pharmacists no definite rank suited to their educational attainments.

### MASSAGE AT MANCHESTER.

An important addition to Manchester's institutions will be made when the transformation of Heaton Park into a large massage establishment for 4,000 wounded is completed. The question of treating joints which have been injured by shrapnel is one to which increasing attention is being given, and an apparatus for electrical treatment, together with a Dowsing radiant heat installation, will probably form part of the equipment. A Canadian surgeon is understood to have arrived already at Heaton Park, and if the work justifies expectation, Manchester is likely to become a permanent centre for dealing with the disabled and maimed among the wounded. The massage department in connection with St. Bartholomew's Hospital shows what can be done in this direction, and a properly equipped centre of sufficient dimensions in the North should prove a valuable accession to the special hospitals of the country.

### FIREGUARDS IN HOSPITALS.

A burning fatality at the Ancoats Hospital, Manchester, which formed the subject of a Coroner's inquiry recently, draws attention once again to the importance of fireguards in public institutions. A nurse in another hospital in the Manchester district lost her life not very long ago through her clothing becoming ignited at an open fire-grate, and in the present case Margaret Alma

Smith, aged twenty-one years, a housemaid at Ancoats Hospital, was the victim. Along with another employee at the institution she was looking at a book of Christmas cards in the kitchen of the hospital when the cotton dress she was wearing caught fire, and, although she was speedily wrapped in blankets, she suffered terrible burns. It was stated that no guard was provided for the fire, and Mr. C. W. W. Surridge, the Acting Coroner, said the wonder to him was that fatalities of this sort were not more common. He thought it would be wise for fireguards to be provided, because it seemed to him there was always a danger of girl employees' skirts catching fire. In returning a verdict of "Accidental death," the jury added a recommendation that fireguards should be provided in all public institutions, and Mr. Surridge said the recommendation would be forwarded to the authorities of the Ancoats Hospital.

### IS IT A WASTE OF PUBLIC MONEY?

The Wandsworth Board of Guardians have decided to call the attention of the War Office to what they describe as "an apparent waste of public money." It appears that Dr. F. W. Abbott, the medical officer at the Tooting Home for the Aged Poor, is in receipt at the present time of a salary of £165 per year from the Guardians, but as the military authorities have taken over that institution his services are not being utilised by them, whilst, at the same time, they are employing a large number of civilian doctors at a guinea a day. The Guardians feel that, having to pay Dr. Abbott's salary for which he is performing no services in return, there is a waste of public money that could well be stopped. They have a further grievance against the same military authorities and their own medical superintendent at St. James's Infirmary—Dr. W. L. Maccormac. The doctor obtained permission from the Guardians to visit daily the military hospital at the Tooting Homes, when it was generally understood that he would give his services gratuitously. The Guardians have now learnt that Dr. Maccormac is receiving the same daily fee as the civilian doctors, which with his salary under the Board brings his income up to £1,020 a year. The Guardians' declared grievance is that they have no power to alter any arrangements made by the military authorities in the two cases referred to.

### A WELL-MERITED INCREASE OF SALARY.

The Lambeth Board of Guardians have decided to grant an increase of salary to Dr. G. F. Stebbing, first assistant medical officer at their infirmary. On the outbreak of the war, Dr. Stebbing was accepted for service as a surgeon in the Royal Navy, but in April last, in response to the request of the Guardians, the Admiralty released him from service in order that he might resume his duties at the infirmary. Whilst serving in the Navy he received a salary much in excess of that he now receives at the infirmary, and his return to the service of the Board involved a financial sacrifice on his part. His present salary is £275 per year, together with emoluments, and, therefore, in view of the special circumstances related above, the Board has resolved to increase his salary to £400 per annum, with emoluments, for the period of the war. This increase will only place Dr. Stebbing on the same scale of salary as is paid by adjoining Boards of Guardians to medical officers occupying similar positions.

### THE EXTRA SERVICES OF SECRETARIES.

We are glad to note a statement by Mr. J. C. Warner, chairman of the committee of management of the Royal Hampshire County Hospital, Winchester, at the recent quarterly court, with reference to the extra responsibilities which had fallen on the shoulders of Mr. A. D. White, the secretary, and the matron since the outbreak of war. After remarking that, although 1,500 soldiers had been assisted, the reception and treatment of civilians had in no way been curtailed, Mr. Warner said that the work of the responsible officers, the secretary and the matron, had increased enormously, and when the time came for the annual report to be laid before the governors, the increased numbers of patients per day, of operations, and of casualties would be apparent. It was in the minds of the committee to recognise these extra services in a way still under consideration. It will be interesting to see the committee's decision; but in the meantime we welcome their appreciation of Mr. White's heavy additional labours, and do not hesitate to recommend to other boards of management the desire of the committee of the Royal Hampshire County Hospital fitly to recognise the secretary's and matron's recent burdens. Hospital secretaries have done wonders since the war, and instances have been all too few of adequate recognition. The general rate of secretaries' salaries is too low, while the war work has shown the great ability needed for administrative posts.

### THIS WEEK'S DRUG MARKET.

There are as yet few signs of any falling off in the volume of business, but as the end of the year approaches the drug market will no doubt become quieter. But for the present it remains active and prices generally continue to advance. The extremely high value of ipecacuanha is well maintained, and there seems little ground to hope for any decline in price in the near future, available supplies being so small and the demand for emetine being so large. Thymol is again dearer. Business in quinine is quite limited, the excitement having completely subsided. Salicylic acid and sodium salicylate are again dearer. There has been no further advance in the price of phenacetin, but it is probable that the highest figure has not yet been reached. Phenazone is again dearer. There has been a further rise in quotations for boric acid. Senna-leaves are fetching higher prices. Opium is unchanged. There is a likelihood of an advance in the price of cocaine, and a larger business is being transacted in this drug.

